# Fatal Allergic Reaction to Gadolinium Contrast

**DOI:** 10.7759/cureus.42455

**Published:** 2023-07-25

**Authors:** Som N Chalise, Elizabeth Palmer, Vikas Pathak

**Affiliations:** 1 Pulmonary and Critical Care, Riverside Health System, Newport News, USA; 2 Pulmonary and Critical Care, Riverside Health System, Yorktown, USA

**Keywords:** mri contrast, disseminated intravascular coagulation, anaphylactic shock, severe allergic reaction, gadolinium based contrast agent

## Abstract

Gadolinium-based contrast agents (GBCA) have been used to enhance the sensitivity and specificity of disease diagnoses. They have excellent safety profiles. However, rare adverse events may happen. We present a case of severe fatal allergic reaction to GBCA in a 35-year-old patient.

## Introduction

Gadolinium-based contrast agents (GBCA) were first used for magnetic resonance imaging (MRI) in the 1980s in order to enhance image sensitivity and specificity of disease diagnoses [1]. GBCAs have been used with MRI in over 100 million patients in the last 25 years [2], and have an excellent overall safety record [3]. In this report, we document the complications and, ultimately, a fatal outcome of severe allergic reaction to GBCA following an outpatient MRI.

## Case presentation

The patient was a 35-year-old Asian American female with a medical history significant for Hashimoto’s disease diagnosed at age 25, now with hypothyroidism, essential tremor, and generalized anxiety disorder. Her only known allergy was to chlorhexidine which resulted in localized skin rash. As an outpatient, she had been having waxing and waning symptoms of blurry vision, decreased sensation in the upper extremities, and subjective feelings of an unsteady gait for a few months. She was referred by her primary care physician for an outpatient MRI of the brain with and without gadolinium contrast and an MRI of the cervical spine without contrast.

For the imaging study, the patient received intravenous (IV) 8mL of gadobutrol, a GBCA. All imaging studies were completed, and at the end of the procedure, she developed sudden-onset shortness of breath, chest heaviness, diaphoresis, diarrhea, nausea, and vomiting. She did not have any known history of previous gadolinium exposure. She was then transported to an urgent care where she was found to be hypoxic with oxygen saturations in the upper 70s. She was started on supplemental oxygen and given diphenhydramine 25 mg IV, dexamethasone 10 mg IV, famotidine 20 mg IV, and nebulized albuterol/ipratropium bromide. Shortly after her arrival, the patient was transferred to a full-service emergency department (ED). En route, she was given epinephrine 0.3 mg intramuscularly (IM) for hypotension.

On arrival at the ED, the patient was in significant respiratory distress without angioedema, skin rash, or any wheezing on physical exam. Her initial vital signs showed a heart rate of 129 beats per minute, blood pressure of 68/43 mmHg, respiratory rate of 24, temperature of 100.6*F, and oxygen saturation of 83% on a non-rebreather mask. She received two additional doses of 0.3 mg IM epinephrine, 25 mg IV diphenhydramine, and 100 mg IV methylprednisolone, and was then started on an epinephrine infusion. A stat chest radiograph revealed evidence of diffuse pulmonary edema. She was placed on non-invasive ventilation for respiratory distress however became increasingly encephalopathic and required endotracheal intubation. Blood cultures were obtained, and she was empirically started on vancomycin, piperacillin-tazobactam, and azithromycin.

The patient was transferred to the medical intensive care unit in shock, on epinephrine and phenylephrine infusions. Her hypotension progressively worsened, and within four hours of presentation, she was on maximum doses of epinephrine (10 mcg/min), norepinephrine (30 mcg/min), phenylephrine (180 mcg/min), and vasopressin (0.03 units/min), as well as stress-dose hydrocortisone. Despite hemodynamic support, she remained hypotensive with systolic blood pressures in the 50s-60s via arterial line. A preliminary diagnosis of anaphylactic shock due to an allergic reaction to gadolinium contrast was made. She had mixed metabolic and respiratory acidosis with lactic acidemia due to profound shock, and respiratory acidosis due to ongoing difficulty with ventilation. She was started on a bicarbonate drip to help support her hemodynamics due to profound acidosis with a pH of 6.6. Pertinent lab values are summarized in Table 1 below. She remained profoundly encephalopathic without any sedation. She had no cough, gag, corneal, or deep tendon reflexes. Her pupils were fixed and dilated although she had spontaneous breaths.

**Table 1:** Pertinent laboratory values summary WBC: white blood cell count; k/mcL: kilo per microliter; Hb: hemoglobin; gm/dL: gram per deciliter; PT: prothrombin time; sec: seconds; INR: International normalized ratio; aPTT: activated partial thromboplastin time; mg/dL: milligram per deciliter; mmol/L: milli mol per liter; AST: aspartate aminotransferase; ALT: alanine aminotransferase; ALP: alkaline phosphatase; IU/L: International unit per liter; T.Bi.: total bilirubin; CK: creatine kinase; mmol/L: millimol per liter.

	Initial labs	4 hours	7 hours	8 hours	10 hours	Reference values
WBC	30.7	38.9			13.1	4.8-10.8 k/mcL
Hb	20.2	18.5			4.1	14-18 gm/dL
Platelets	362	225			98	150-450 k/mcL
PT				>114		10-12.6 sec
INR				>12.5		0.9-1.1
aPTT				>200		20-35 sec
Fibrinogen				<25		200-400 mg/dL
Bicarb	16	12	7		14	22-32 mmol/L
BUN	18	18	18		17	7-24 mg/dL
Creatinine	1.42	2.32	2.44		2.45	0.61-1.24 mg/dL
AST	34		386		339	7-37 IU/L
ALT	21		573		505	7-41 IU/L
ALP	76		130			32-129 IU/L
T. Bi.	0.9		0.5		0.9	0.1-1.5 mg/dL
CK			163			49-397 U/L
Lactic acid			>11.1		>11.1	<2 mmol/L
pH	7.05	6.60		6.80		7.35-7.45
pCO2	60.4	67.7		48.8		35-45
Urinalysis	Negative for infection					
Blood culture	No growth					
Covid19	Negative					

Electroencephalogram revealed moderate diffuse slowing with no evidence of seizure activity. Electrocardiogram showed normal sinus rhythm with sinus tachycardia without any acute ST-T wave changes. Echocardiogram was normal except a small pericardial effusion. She became anuric with worsening acute renal function (creatinine increased from 1.42 to 2.45 mg/dL). There was blood noted to be oozing from the femoral arterial line and internal jugular vein central line sites. She had blood mixed secretions from her nares and orogastric tube. Coagulation studies showed that she was in profound disseminated intravascular coagulation (DIC), with a drop in platelet counts to 98 k/mcL, fibrinogen <25 mg/dL, D-dimer > 35.20 mg/L, prothrombin time (PT) >114 sec, INR >12.53, and partial thromboplastin time (PTT) >200 sec. She received fresh frozen plasma, but while awaiting the cryoprecipitate and blood products, the patient had a pulseless electrical activity (PEA) cardiac arrest within 17-18 hours of receiving GBCA. Resuscitation with advanced cardiac life support protocol was performed. Return of spontaneous circulation was achieved briefly several times. However, due to her very poor prognosis, the patient’s family at the bedside decided to discontinue aggressive care and direct the goals of care toward making her comfortable. An autopsy was performed which revealed pulmonary edema, bilateral pleural effusions, ascites, small pericardial effusion, and hepatic congestion, but did not reveal any other specific findings. All culture data were finalized as no growth.

## Discussion

GBCA results in the enhancement of tissue images, thereby enhancing the sensitivity and specificity of diagnostic images [2]. These contrast agents have an excellent safety record, except in patients with severe renal failure where a rare but serious condition called nephrogenic systemic fibrosis may result [3]. Although there is a potential to have an allergic reaction to any substance, reactions to GBCAs are considered rare, occurring with 0.004% to 0.7% of exposures. Additionally, the majority of reactions are classified as mild in nature, although severe reactions may occur 0.001% to 0.01% of the time [4]. In a retrospective study by Jung et al., the incidence of immediate hypersensitivity reaction to an MRI contrast agent was reported as 0.079%, and the recurrence rate of hypersensitivity reaction was 30% in patients with a previous reaction [5].

In our case, a young patient in relatively good health received a GBCA to investigate neurologic symptoms. Shortly afterward, she developed cardiopulmonary collapse and profound DIC, ultimately resulting in death within less than 24 hours from exposure to a GBCA. There were no sources of infection and culture data remained negative. Echo did not show evidence of heart failure. Her autopsy did not reveal anything specific as a cause. With the temporal association of symptom onset following the patient receiving contrast, and with no other causes noted clinically, radiologically, within the laboratory data, or through autopsy, it was concluded that her cause of death was due to a severe allergic reaction to a GBCA. It is interesting to note that our patient did not have a cutaneous reaction, which usually accompanies anaphylactic shock. Our patient also suffered from DIC during her severe allergic reaction. It has been found from animal models that platelet-activating factor (PAF) is involved in both DIC and anaphylaxis. DIC was prevented in animals if pretreated with anti-PAF [6]. It may be that PAF may stimulate IL-1 and tumor necrosis factor (TNF)-alpha.

## Conclusions

Even though it is very rare to have an allergic reaction from an MRI contrast agent, if it happens, it may progress rapidly to severe anaphylaxis, DIC, and potentially death. The radiology department and the treating physician should be aware of this rare reaction and start the necessary management if the event occurs. More data is still needed in this area to determine the frequency of these reactions and if any common risk factors exist.
